# Toward Cyborg: Exploring Long-Term Clinical Outcomes of a Multi-Degree-of-Freedom Myoelectric Prosthetic Hand

**DOI:** 10.34133/cbsystems.0195

**Published:** 2025-03-18

**Authors:** Yuki Kuroda, Yusuke Yamanoi, Hai Jiang, Yoshiko Yabuki, Yuki Inoue, Dianchun Bai, Yinlai Jiang, Jinying Zhu, Hiroshi Yokoi

**Affiliations:** ^1^ Graduate School of Informatics and Engineering, University of Electro-Communications, Tokyo, Japan.; ^2^ Department of Electrical Engineering, Faculty of Engineering, Tokyo University of Science, Tokyo, Japan.; ^3^ School of Electrical Engineering, Shenyang University of Technology, Shenyang, China.; ^4^ Center for Neuroscience and Biomedical Engineering, University of Electro-Communications, Tokyo, Japan.; ^5^ Beijing Advanced Innovation Center for Intelligent Robots and Systems, Beijing, China.

## Abstract

Recent advancements in robotics and sensor technology have facilitated the development of myoelectric prosthetic hands (MPHs) featuring multiple degrees of freedom and heightened functionality, but their practical application has been limited. In response to this situation, formulating a control theory ensuring the hand dexterity of highly functional MPHs has garnered marked attention. Progress in this field has been directed toward employing machine-learning algorithms to process electromyogram patterns, enabling a broad spectrum of hand movements. In particular, the practical application of 5-finger-driven MPHs with such control functions to real users remains limited, and their attributes and challenges have not been thoroughly examined. In this study, we developed a 5-finger MPH equipped with pattern recognition capabilities. Through a long-term clinical trial, encompassing task assessments and subjective evaluations via questionnaires, we explored the MPH’s range of applications. The task assessments revealed an expanded range of achievable tasks as the variety of motions increased. However, this enhanced adaptability was paralleled by a decrease in control reliability. Additionally, findings from the questionnaires indicated that enhancements in task performance with MPHs might be more effective in reducing workplace-related disability than in improving activities in everyday life. This study offers valuable insights into the long-term clinical prospects and constraints associated with multi-degree-of-freedom MPHs incorporating pattern recognition functionality.

## Introduction

Recent advancements in robotics and sensors have made cyborg technology a reality, with myoelectric prosthetic hands (MPHs) serving as a notable example. In particular, advancements such as the development of small high-torque motors [1], compact high-performance microcontrollers [2], and dependable myoelectric sensors [3] have facilitated the manufacture of highly functional MPHs featuring multiple degrees of freedom (DOFs). As a result, constructing a control theory that guarantees the dexterity of highly functional MPHs has become an important issue, and development is proceeding in the direction of machine-learning processing of electromyogram (EMG) patterns to realize a large number of hand motions. Multifunctional MPHs are in high demand for people with upper limb deficiencies and are expected to improve the quality of life of users [4].

However, examples of the application of multi-DOF MPHs to actual users are limited, and their characteristics and associated challenges have not been completely identified. Developing a highly functional MPH and improving its performance based on user feedback is the key to enhancing user convenience. Feedback, especially in the long-term application of MPHs, is the most important for revealing hidden problems in the clinical environment.

Various highly functional MPHs have been developed. For example, commercial multi-DOF MPHs include Bebionic by Ottobock [5] and i-Limb by Össur [6]. Wire-driven multi-DOF MPHs with 13 DOFs are currently in the research phase conducted by Seki et al. [7], and soft neuroprosthetic hands are being studied by Gu et al. [8]. As for high-performance control methods, pattern recognition control methods applicable to multi-DOF control have been developed [9–11] in contrast to methods such as threshold control and proportional control [12], which are primarily applied to single-DOF control.

Studies on the long-term application of such high-performance MPHs include the long-term application of a 5-finger MPH with a commercial pattern recognition function, namely, the COMPLETE CONTROL system developed by Coapt LLC, for more than 8 weeks [13] and a 6-month application of Michelangelo, a commercial MPH [14]. Simon et al. applied multi-DOF MPHs to 4 individuals with upper limb deficiencies over a period of 8 weeks. They found that users tended to use a greater variety of motions for pattern recognition. Studies have also evaluated high-functioning MPHs by interviewing the stakeholders. Franzke et al. [15] interviewed experienced MPH users and therapists and reported that pattern recognition is intuitive but unreliable for everyday use and requires extensive training.

Although feedback can be obtained from these previous studies, the MPH is a device in which various factors, such as the hand’s shape, sensor material, and software performance, have a compound effect, and the operating feel varies considerably depending on the combination used. Therefore, research on the long-term clinical evaluation of high-performance MPHs is still insufficient.

In this study, we developed a 5-finger-driven MPH with a pattern recognition function and applied it to individuals with upper limb deficiencies over the long term. This MPH is lightweight and has a mechanism to release external forces on the fingers, preventing damage and ensuring safe use. It is also equipped with an EMG sensor for robust measurement and a recognition stabilization filter to cope with the limitations in computer performance. We applied this MPH to 2 people with upper limb deficiencies for approximately 3 months and quantitatively evaluated the effects of the long-term application of a multi-DOF MPH with pattern recognition on the users in terms of task performance and subjective evaluations of the degree of disabilities. The purpose of this study was to clarify the range of applicability of the MPH obtained from the results of long-term application and to develop an MPH that is more convenient for the user.

The task evaluation results delineated the spectrum of achievable tasks. Additionally, the findings indicated that augmenting the variety of motion patterns enhances task performance. However, it is noted that training is necessary to discern the suitable motion pattern for each task. Furthermore, the questionnaire evaluation results indicated that enhancing task performance with MPHs might be more effective in alleviating disability in the workplace than in everyday life.

## Five-Finger-Driven MPH

**Fig. 1.**
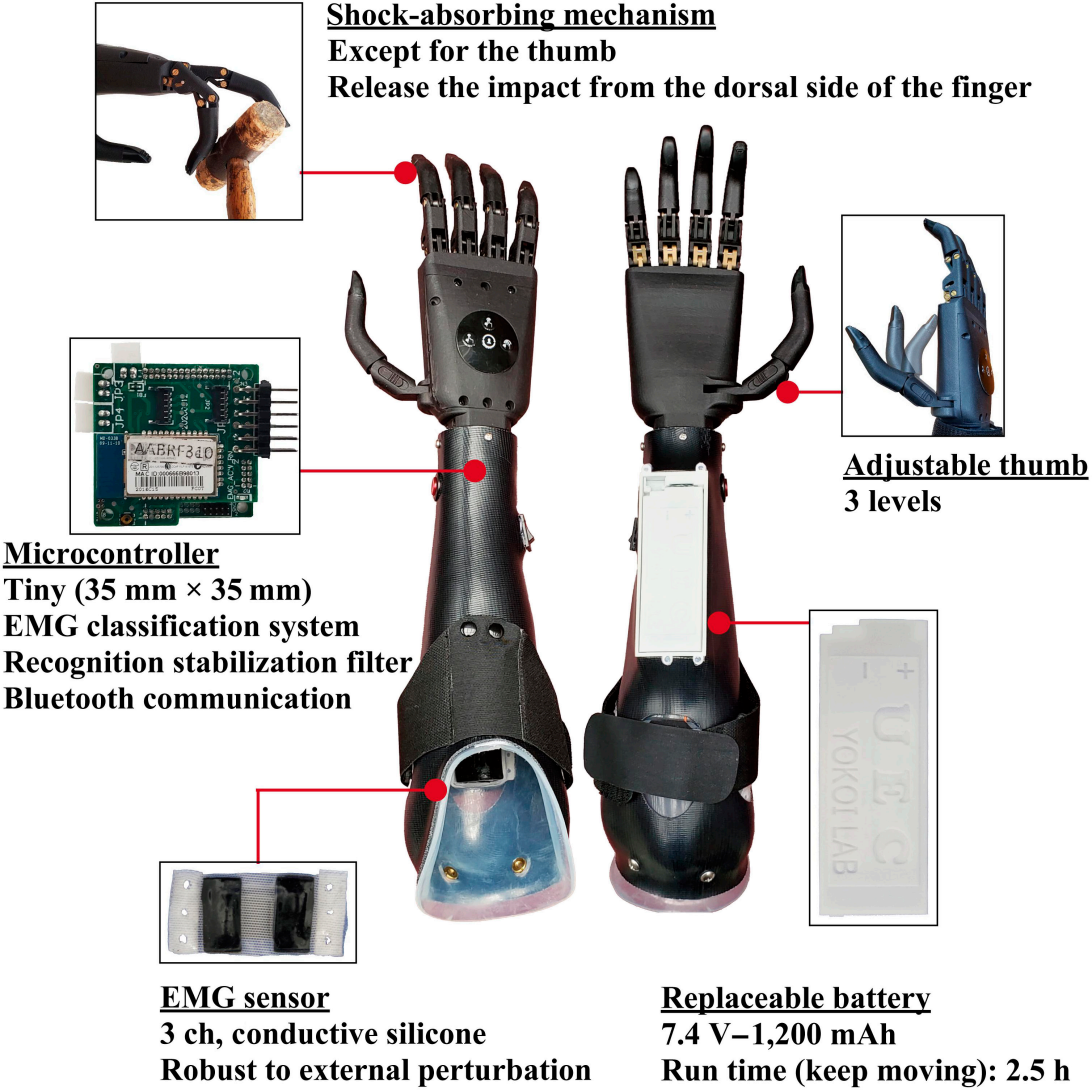
Overview of the BIT-UEC-Hand. EMG, electromyogram; 3 ch, 3-channel.

The BIT-UEC-Hand developed in this study consists of a hand, a controller, an EMG sensor, a battery, and a socket [16,17]. The hand was developed in a joint research project between the University of Electro-Communications (UEC) and the Beijing Institute of Technology (BIT). The BIT-UEC-Hand developed in collaborative research is equipped with a controller developed in the Yokoi Laboratory of the UEC. The controller can communicate with the tablet application to check the myoelectric signal information and acquire teacher data for pattern recognition control. In addition, this MPH was registered as a ready-made part in 2022 by the Ministry of Health, Labour and Welfare. Therefore, it can be purchased in Japan with the assistance of local governments. Fig. 1 summarizes the main functions of the BIT-UEC-Hand.

### Hardware

#### Five-finger-driven electric prosthetic hand

As the weight of a human hand is approximately 400 g [18], the weight of several commercial multi-DOF MPHs is in the range of 400 to 500 g, which appears to be sufficient in terms of weight. However, the muscles, fascia, tendons, bones, and other structures that make up the human body distribute this weight to the upper limb to lighten the load [19–21]. When an MPH is applied to a person with an upper limb deficiency, the MPH and residual limb are entirely independent; thus, the load of the MPH and the grasping object is applied to the residual limb. Therefore, the MPH needs to be as light as possible.

The hand is mostly made up of nylon, with an aluminum alloy at some joints and rubber at each fingertip. The weight of the hand is approximately 330 g, which is 100 to 200 g lighter than the existing commercial 5-finger electric prosthetic hands. This is expected to reduce the load on the user. The hand has 11 joints in total. The active DOFs are 1 for each finger (thumb: carpometacarpal joint; others: metacarpophalangeal joint) for 5 DOFs. As passive DOFs, the metacarpophalangeal joint of the thumb can be adjusted in 3 steps, and the wrist can be rotated 360°. A linear motor (thumb: LAS10-23D; others: LA10-21D; Beijing Inspire Robots Technology Co., Ltd.) is used as the actuator, and the maximum pinch force of the fingertips was approximately 12 N. Fingers other than the thumb have a mechanism to release external forces. Therefore, they can cope with impacts from the dorsal side of the fingers, which is expected in daily life owing to the lack of somatosensory and body possession senses, thereby providing a high degree of safety.

The design concept of this hand is described in detail by Wang et al. [17]. Three links generate the trajectory of the 4 fingers. This underactuated connecting rod mechanism provides the fingers with an underactuated grasping ability. In other words, the fingers can be bent along the object, ensuring at least 2-sided contact between the fingers and the object and, thus, the stability of the grasping motion.

#### Controller

Because the controller must be built into the socket, it is compact and lightweight, with dimensions of 35 mm × 35 mm × 14 mm, excluding the connector, and a weight of 28 g. The lower part is equipped with 200-MHz SH72546R (Renesas Electronics Corporation), which has sufficient calculation speed for real-time EMG signal analysis, and the upper part is equipped with a connector for connecting the sensor and the motor and a Bluetooth module (RN42-I/RM, Microchip Technology Inc.) used for communicating with the tablet. SH72546R has an electrically erasable programmable read-only memory (EEPROM), which can store the trained classifier model’s and the hand’s parameters.

#### EMG sensor

We used the EMG sensor previously developed by our research group [3]. This EMG sensor comprises an electrode part that contacts the skin and a sensor amplifier part that amplifies minute myoelectric potentials. The electrode part is made of conductive silicone, which enables a more stable myoelectric measurement against an external force than dry electrodes generally used for commercial MPHs. Conductive silicone consists of a contact electrode that contacts the skin and a base electrode between the contact electrode and a gold-plated wire. The gold-plated wire is connected to the sensor amplifier. The conductive silicone was fabricated by mixing silicone (TSG-E30, Tanac Co., Ltd., Japan) and carbon black (EC600JD, Lion Specialty Chemicals Co., Ltd., Japan). The carbon content of the contact electrode is 2.4%, and that of the base electrode is 4%. The sensor amplifier section contains a differential amplifier circuit, a 50-Hz notch filter to mitigate 50-Hz electromagnetic noise from commercial power sources in eastern Japan, a 10-Hz high-pass filter, and a 400-Hz low-pass filter to effectively acquire the frequency range of EMG signals.

#### Battery

The battery is a high-power lithium polymer battery that can drive multiple motors. Even when the hand constantly runs, it can be used for about 2.5 h. In addition, the cartridge system allows immediate battery replacement if the battery runs out. The battery can be recharged with the charger that we have developed.

#### Tablet application

**Fig. 2.**
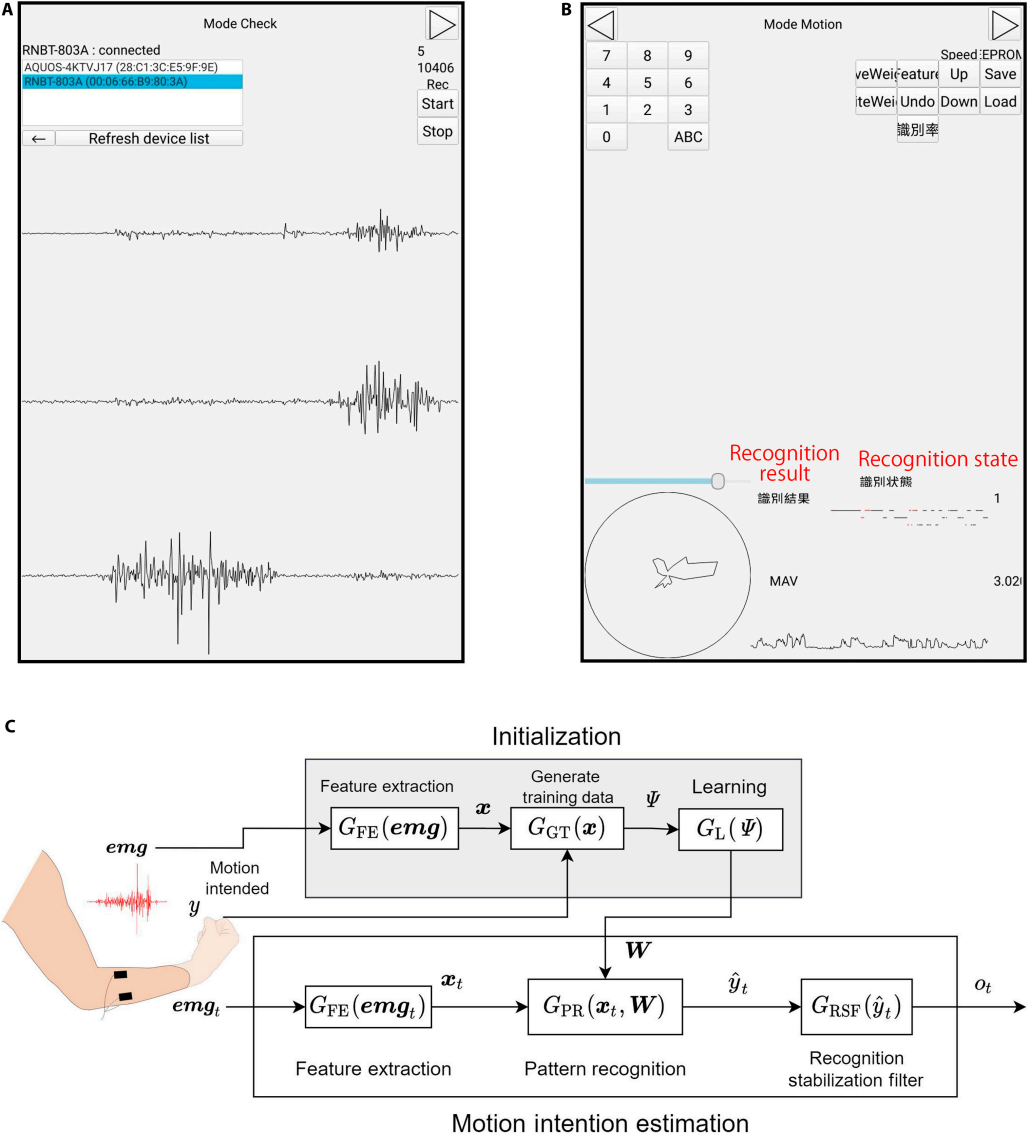
(A and B) Screenshots of the tablet application. The Japanese-language indications relevant to this study have been translated into English and highlighted in red. (A) Mode check: confirmation and measurement of raw EMG signals are possible. The upper section of the screen contains buttons for selecting the microcontroller and establishing Bluetooth connectivity with it. The 3 waveforms displayed at the bottom are raw EMG data. Each waveform represents a raw EMG signal from channel 1 to channel 3 of the 3-channel EMG sensor. (B) Mode motion: allows the inspection of features on a radar chart, including the mean absolute value (MAV) of the EMG, labeling of teacher data, and recognition results. The radar chart is displayed in a circle at the lower left section, with 3 circular sector sections illustrating the values of the 3-channel feature data. The recognition result is indicated by a number (e.g., “1”) on the right side of the figure, corresponding to each motion. The value “3.020” in the lower right corner denotes the MAV, accompanied by the MAV waveform displayed in the lower right section. The upper part of the tablet screen contains buttons for labeling teacher data. (C) Classification of EMG signals.

The utilized tablet application was “Signal Viewer”, which was developed by our research group. Through Bluetooth communication, the controller connects to Signal Viewer, enabling users to inspect raw EMG signals, feature values represented in radar charts, and identification outcomes. Furthermore, when employing pattern recognition to control the MPH, users can label the teacher data by pressing the appropriate motion button, initiating the sampling process conducted by the controller. The “EMG classification system” section provides an extensive description of the process for labeling the teacher data. Screenshots of the tablet application can be found in Fig. 2A and B.

### Software

#### EMG classification system

Using pattern recognition based on the EMG of the user’s forearm, the hand component is controlled by identifying the user’s intention to move. An overview of the EMG classification system is shown in Fig. 2C. Experiments on nondisabled individuals have shown that this system is capable of discriminating 8 motions with an average recognition rate of 83.8% [16]. In addition to this system, a motor switch is mounted on the socket’s wrist that allows the hand movement to be fixed manually to address the reliability problem of pattern recognition control, as reported by Franzke et al. [15].

The system comprises 2 primary processes: initialization and motion intention estimation. First, we delineate the initialization phase. Following the measurement of EMG emg, it undergoes conversion into a feature x format conducive for discerning motion intentions, achieved through the feature extraction function $G_{\mathrm{FE}}$.

During this stage, the simultaneous measurement of the motion intention y associated with the generated EMG emg occurs. The determination of y involves pressing a button on the tablet application, signifying the motion intention and subsequently triggering EMG measurement. Subsequently, the teacher data $\psi =\left(x,\;y\right)$ are generated by the teacher data generation function $G_{\mathrm{GT}}$. These teacher data are buffered by $G_{\mathrm{GT}}$ until a specific number is accumulated, forming a set of teacher data denoted as Ψ—a process termed labeling. Utilizing Ψ, the statistical learning function $G_{L}$ establishes the relationship between the EMG and motion intention, deriving the parameter W for the pattern recognition function $G_{\mathrm{PR}}$.

Moving to the motion intention estimation process, the EMG signals ${\mathrm{emg}}_{t}$ measured by the EMG sensor at time t are similarly transformed into a feature value $x_{t}$ by the feature extraction function $G_{\mathrm{FE}}$, mirroring the initialization process. The pattern recognition function $G_{\mathrm{PR}}$ then outputs ${\hat{y}}_{t}$, which is the predicted value of motion intention. In this case, ${\hat{y}}_{t}$ is selected from the set of motion intentions Y learned at initialization. ${\hat{y}}_{t}$ is inputted into the recognition stabilizing filter $G_{\mathrm{RSF}}$, which outputs the motion intention $o_{t}$. The recognition stabilization filter refers to the time-series data of the estimated motion patterns stored in a ring buffer for a certain period. It determines the output based on the proportion of estimated motion patterns in the time series [22]. Assuming that the ring buffer stores the past N outputs, the ring buffer set $B_{t}$ at time *t* is $B_{t}=\left({\hat{y}}_{t-N},\;{\hat{y}}_{t-N+1},\;\ldots ,\;{\hat{y}}_{t},\;{\hat{y}}_{t}\right)$. In this case, the recognition stabilization filter $G_{\mathrm{RSF}}$ performs the following process:

$$B_{t}⋃{\hat{y}}_{t}\to B_{t}$$ (1)

$$B_{t}\backslash {\hat{y}}_{t-\left(N+1\right)}\to B_{t}$$ (2)

$$o_{t}=\underset{i\in Y}{\text{arg}\;\text{max}}f_{i}\left(B_{t}\right)$$ (3)

where $f_{i}$ in Eq. (3) is a function for calculating the number of class i in the buffer.

#### System settings

The specific configurations for each function employed in this study are detailed in Table 1. As shown in the table, within the feature extraction function $G_{\mathrm{FE}}$, the EMG data, sampled at 2,000 Hz, underwent conversion into frequency components via fast Fourier transform. Among the 256 frequency components in each channel, 8 feature values were extracted. The EMG’s frequency band is conventionally considered to span from 0 to 500 Hz [23], serving as a reference for extraction. Nevertheless, the literature indicates that the predominant EMG energy resides within the range of 30 to 150 Hz [23] or 20 to 150 Hz [24], with higher power observed between 100 and 200 Hz [25]. Consequently, our extraction primarily focused on these frequency bands. Subsequently, the frequency components preceding and following each extracted feature value (5 points in total) were aggregated and averaged. This process facilitated the amalgamation of elements from the EMG frequency band into the feature vector.

**Table 1.** Experimental conditions

Measurement	
Sampling frequency (Hz)	2,000
Quantization bit rate (bit)	12
Filter (hardware)	50-Hz notch filter
	1- to 1,000-Hz band-pass filter
Filter (software)	50-Hz high-pass filter
Potential range (V)	−2.5 to 2.5
Feature extraction function: $G_{\mathrm{FE}}$	
Method	FFT
Period (ms)	10
Number of samples	256
Overlapping samples	236
Window function	Hann window
Extracted frequencies (Hz)	23.4375, 46.875, 70.3125, 93.75, 140.625, 187.5, 250.0, 312.5
Smoothing (points)	5
Pattern recognition function: $G_{\mathrm{PR}}$	
Method	ANN
Number of input layer neurons	*D* = 24
Number of hidden layer neurons	32
Number of output layer neurons	8
Activation function	Sigmoid
Learning function: $G_{L}$	
Method	Gradient descent
Learning rate	0.01

FFT, fast fourier transform; ANN, artificial neural network

The pattern recognition function utilized in this study was the artificial neural network (ANN). In recent years, recognition methods based on deep learning leveraging large datasets have emerged [26,27]. While these approaches demonstrate the capability to accurately recognize various motion types, they necessitate extensive training periods on high-performance computers and considerable time to acquire adequate teacher data. As previously mentioned, the practical application of MPHs demands smaller and lighter controllers to safeguard the controller within the socket and alleviate the strain on the user’s residual limb. Waiting for extended learning completion times or prolonged data sampling periods is impractical in such scenarios. Hence, ANN was chosen for this study due to its established efficacy demonstrated in prior research [10,16,28].

### Sampling teacher data

As delineated in the “Software” section, preliminary sampling of EMG data emg and motion intention y is imperative for executing pattern recognition control. This motion intention correlates with the EMG data through the tablet application by pressing a button associated with the intended motion, as expounded in the “Tablet application” section. Specifically, upon executing a specific motion during using the tablet application, the user links it to the EMG data by pressing the corresponding motion’s button. The process of sampling teacher data unfolds in the following steps:

1. The user wears the MPH.
2. The user performs a specific muscle activity, linking this signal with the EMG data emg and motion intention y by pressing the corresponding motion’s button on the tablet application (the numerical buttons depicted in Fig. 2B).
3. Step 2 is reiterated for all motions slated for learning.

In instances where upper limb amputees lack a hand, performing actual hand motions becomes unfeasible. Nevertheless, individuals with acquired upper limb amputations often retain the relevant muscles and can manifest distinct muscle contraction patterns through mental visualization of specific motions. In this study, given that the participants had acquired upper limb amputations, we collected teacher data by instructing them to engage in muscle contractions while mentally envisioning the specific motions using their residual limbs. For congenital amputees, a potential approach to implementing this control technique involves executing the same motions using the unaffected side while gathering the teacher data [29,30].

## Long-Term Clinical Evaluation of the BIT-UEC-Hand Applied to People with Upper Limb Deficiency

Clinical evaluation of the BIT-UEC-Hand was conducted with the cooperation of 2 participants with an acquired upper limb deficiency over a period of approximately 3 months. Participant A was the user of a 2-DOF MPH with pattern recognition control and had been using it for more than 10 years at the time of the study. His symptoms included an amputation of the right forearm. Participant B was the user of a single-DOF MPH with proportional control and had been using it for about 5 months. The symptom was an amputation of the right forearm. The purpose and content of the experiment were fully explained to the participants in advance, and their consent to participate in the experiment was obtained. This experiment was conducted with the approval of the University of Electro-Communications Ethics Committee (No. 10006) and Tokai University Hospital Ethics Committee (No. 14R-199). Furthermore, all procedures adhered to the principles outlined in the Declaration of Helsinki.

**Fig. 3.**
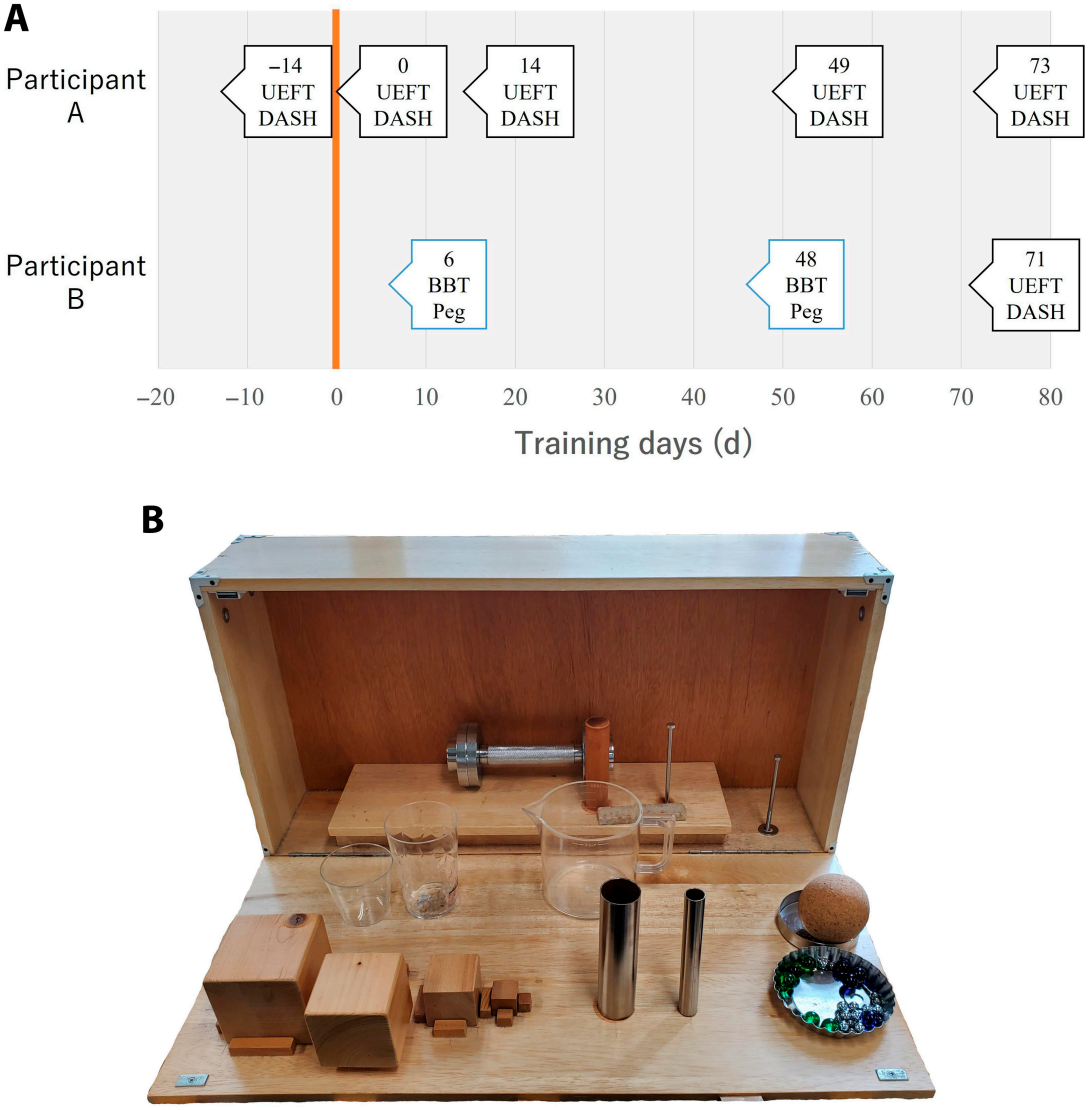
(A) Experiment schedule. Day 0 (orange line) is the day that the participant started using the BIT-UEC-Hand in each household. The position of the arrow corresponds to the day on which the evaluation was performed. The text in the squares indicates the day and type of assessment conducted. (B) Items for the Upper Extremity Function Test (UEFT). DASH, Disability of the Arm, Shoulder, and Hand; BBT, Box and Block Test; Peg, Large Peg Pull Test.

The 2 evaluation methods included task evaluation and questionnaire evaluation, and the evaluation schedule for each of the 2 participants is shown in Fig. 3A. The number of days for each evaluation was counted from day 0 when the BIT-UEC-Hand was confirmed to be used without any problems and the use of the MPH was started in each home. Notably, each evaluation center was different due to factors such as the participant’s residence and the evaluation tests that differed depending on the centers maintained by the evaluation center. However, regarding the evaluation tools, consistency in assessment is maintained even when the evaluation centers differ, as the same evaluation criteria and tools are employed.

### Task evaluation

Task evaluation is one of the most widely used evaluation methods for evaluating the performance of MPHs. Representative evaluation methods include the Southampton Hand Assessment Procedure [31], the Box and Block Test (BBT) [32,33], and the Action Research Arm Test [34]. However, these methods depend on task execution time, making it difficult to evaluate multi-DOF MPHs, which require time to drive the hand, or in the early stages of training. Assessment of Capacity for Myoelectric Control (ACMC) [35,36] does not depend on task performance time. The ACMC scores are based on 30 items related to gripping, holding, releasing, and bimanual coordination in daily activities, not on specific tasks. Therefore, it is difficult to perform the test without specialized knowledge. Hence, the Upper Extremity Function Test (UEFT) [37] was employed in this study to evaluate upper limb function in a defined task without temporal evaluation. The evaluation center near participant B’s residence did not possess the UEFT, and the UEFT evaluation was conducted only during the latter part of the assessment period at a different evaluation center. To assess the MPH’s performance closer to participant B’s residence, BBT and the Large Peg Pull Test (Peg), which evaluate the time required for task completion, were employed as supplementary assessments. In the task evaluation, the participant selected the movement patterns that were stable and controllable from the following: resting, full-finger grip, open, 2-finger pinch, and 3-finger pinch. Therefore, the number of movements used depended on the stability of control on that day.

#### Upper Extremity Function Test

The UEFT is an upper extremity functional assessment method for hemiplegic patients [37]. It is suitable for the assessment of arm mobility in addition to hand evaluation. The results of a 32-item task to handle the 17 objects shown in Fig. 3B are evaluated. The UEFT evaluates tasks at the following 4 levels: 3 points: the task is completed successfully; 2 points: the task is completed but slowly or very clumsily; 1 point: the task is partially completed; and 0 points: no part of the task can be completed. In this study, however, some of the evaluation and task performance methods were changed such that a person could perform the task using an MPH.

In the pinch evaluation, only one movement is evaluated for each item. When pinching is performed using multiple fingers, each finger is separately assessed (e.g., if the task is successfully completed with a 3-finger pinch, 3 points are given for each item pinching with the index finger and the thumb and for each item pinching with the thumb and the middle finger). Because the BIT-UEC-Hand currently uses only the index finger and the thumb for a 2-finger pinch, the evaluation items for using different fingers for a 2-finger pinch do not make sense. Therefore, this evaluation aimed to assign higher points to cases in which a pinch with more contact points was possible.

Small balls and washers, such as marbles and iron balls, were originally removed from a saucer or a protruding nail; however, in this experiment, they were placed on the palm of the participant’s healthy hand or the desk. In this case, the maximum score was set to 2. The test should be conducted in a sitting position; however, due to the limited range of motion of the MPH, the test was conducted in a standing position when necessary. As for the materials, a clothes iron was substituted with a dumbbell of a similar mass due to availability issues.

#### Box and Block Test

BBT is a measure of manual dexterity [32,33]. The test consists of placing a wooden block measuring 2.5 cm per side in 1 of 2 adjacent boxes, carrying the block one at a time for 1 min, and evaluating the number of pieces moved.

#### Large Peg Pull Test

Peg is used to evaluate manual dexterity [38]. Usually, the number of cylindrical pegs that can be inserted into a board with holes in a certain amount of time is evaluated. However, in this study, we conducted a pullout test instead of insertion, which requires the coordinated action of a normal hand, to measure only the skill of MPH control. The number of pegs that could be pulled out in 1 min was recorded.

### Questionnaire evaluation

#### Disability of the Arm, Shoulder, and Hand

DASH is a self-assessment instrument that evaluates the degree of disability of people with disabilities in their upper limb function in daily life [39]. The Japanese version of DASH, DASH-JSSH [40], was used in this study. The questionnaire consisted of 2 choice items: disability/symptoms, work, and sports/music. Each question is scored on a 5-point scale, with higher scores indicating greater disability. The respondents were asked to answer questions about their symptoms during the last week and imagine how well they could perform the activities they did not actually perform. In this study, participants were instructed to answer questions about their condition when using the BIT-UEC-Hand, and the questionnaire was evaluated.

#### Additional questionnaire

After the clinical experiment, an additional questionnaire regarding the control of the multi-DOF MPH was administered. The questionnaire items are shown in Table 2(a) with the results.

**Table 2.** (a) Results of additional questionnaires and (b) range of application of the multi-DOF MPH

(a)		
Questions (options)	Participant	
	A	B
From how many motion patterns, including resting motions, does it become difficult to use the device in daily life?	4	5
Frequency of relearning (1: relearn every time; 2: relearn frequently; 3: rarely relearn; and 4: never relearn)	2	2
Burden of relearning (1: not at all burdensome [no problem as it is]; 5: quite burdensome [difficult to continue using])	1	3
(b)		
Item	Level of achievement	
Realization of various hand movements	**	
Stable grasping is achieved by increasing the contact points because the 5 fingers grip along the object	***	
The fingers in the way during precision grasping can be flexed and extended to enable grasping in tight spaces	***	
Enables the palm to be extended and to hold things down	***	

Multi-DOF, multi-degree-of-freedom; MPH, myoelectric prosthetic hand

## Results and Discussion

### Task evaluation

**Fig. 4.**
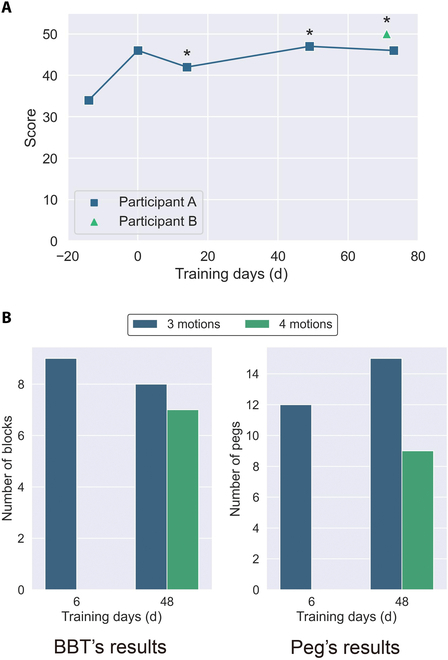
(A) UEFT’s results. Notably, the number of motions used was different for each test. Four motions were evaluated when the * was on the pointer; otherwise, 3 motion controls were used. The fourth motion was a 3-finger pinch in all cases. Day 0 is the day that the participant started using the MPH in each household. (B) BBT’s results and Peg’s results. The fourth motion was a 2-finger pinch.

**Fig. 5.**
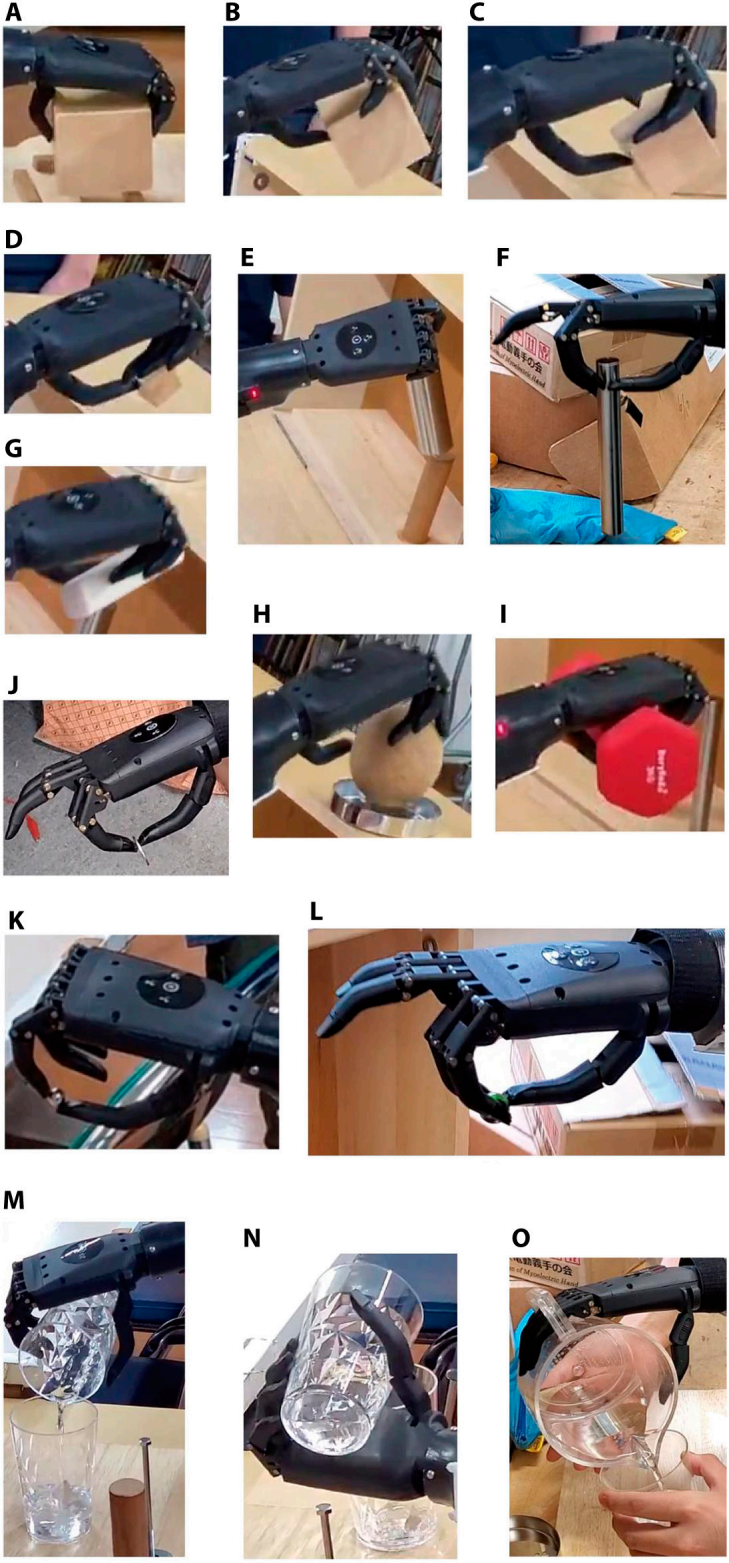
Tasks that could be performed with the UEFT. This table summarizes the actions that resulted in 2 or 3 points that were determined to be feasible by the UEFT. (A) Wooden cube (9 cm); (B) wooden cube (7 cm); (C) wooden cube (5 cm); (D) wooden cube (2.5 cm); (E) large iron pipe (ϕ 38 mm); (F) small iron pipe (ϕ 19 mm); (G) slate (2.5 cm × 1.2 cm × 11 cm); (H) wooden ball (7.5 cm); (I) dumbbell (3 kg, ϕ 28 mm); (J) steel washer (ϕ 78 mm × 2 mm); (K) glass marble (ϕ 17 mm); (L) metal sphere (ϕ 11 mm); pouring water from a glass into glass: (M) pronation and (N) supination; and (O) pouring water from a pitcher into a glass.

A graph summarizing the results of the UEFT is shown in Fig. 4A, and a graph summarizing the actions that were feasible in any trial in the UEFT is shown in Fig. 5. The feasibility was scored as 2 or 3 on the UEFT. Participant A already had a high score from the second trial, indicating that he could become proficient in using the multi-DOF MPH in a short period, even before the start of home training. The third trial, which was the first time that the participant was evaluated with a 4-motion control, showed a slight decrease in the score compared to the previous trial, but the fourth trial showed a score higher than that of the third trial.

Graphs summarizing the results of the BBT and Peg are shown in Fig. 4B. The experiment was conducted with only 3 motions on the sixth day because, at that stage, participant B had not yet acquired proficiency in using the BIT-UEC-Hand and could achieve stable control only with the 3 motions.

Although these results are for reference purposes only due to being obtained from a single trial, the decrease in scores using 4 motions compared to those using 3 motions in both tests indicates a potential negative impact of multiple-pattern control on speed and control accuracy. Pattern recognition necessitates replicating muscle contraction patterns akin to those in the initially recorded teacher data. Presently, the primary cause of control instability is believed to be excessive muscle contraction intensity or alterations in the muscle contraction technique, resulting in reduced control stability [29]. Additionally, Franzke et al. [15] noted that during actual pattern recognition usage, control instability arises from socket movement in relation to the skin, applied pressure, temperature variations, and humidity. The augmentation of pattern numbers is hypothesized to heighten the complexity of reproducing such muscle contraction patterns and amplify the effects of artifacts, potentially contributing to the observed score reduction.

By contrast, participant B, a user of a proportional-control MPH, underwent the evaluation task using 3-motion control from the early stages of training. After 80 d, he surpassed the record of participant A, a user of a pattern-recognition-controlled MPH, on the UEFT, contradicting previous studies that indicated that extensive training was necessary. This result may be attributed to the fact that participant B effectively utilized the motor switches mounted on the MPH system used in this study to fix the hand motion during the experimental process, which might have provided some support for the reliability of the control.

In addition, the UEFT results showed that the participants tended to score higher when identifying the 4 movements. Because UEFT does not have a time limit, it is thought that even when control reliability or task execution speed is low, the score is not affected, and the number of tasks that can be executed can be increased by searching for the most appropriate action.

### Questionnaire evaluation

**Fig. 6.**
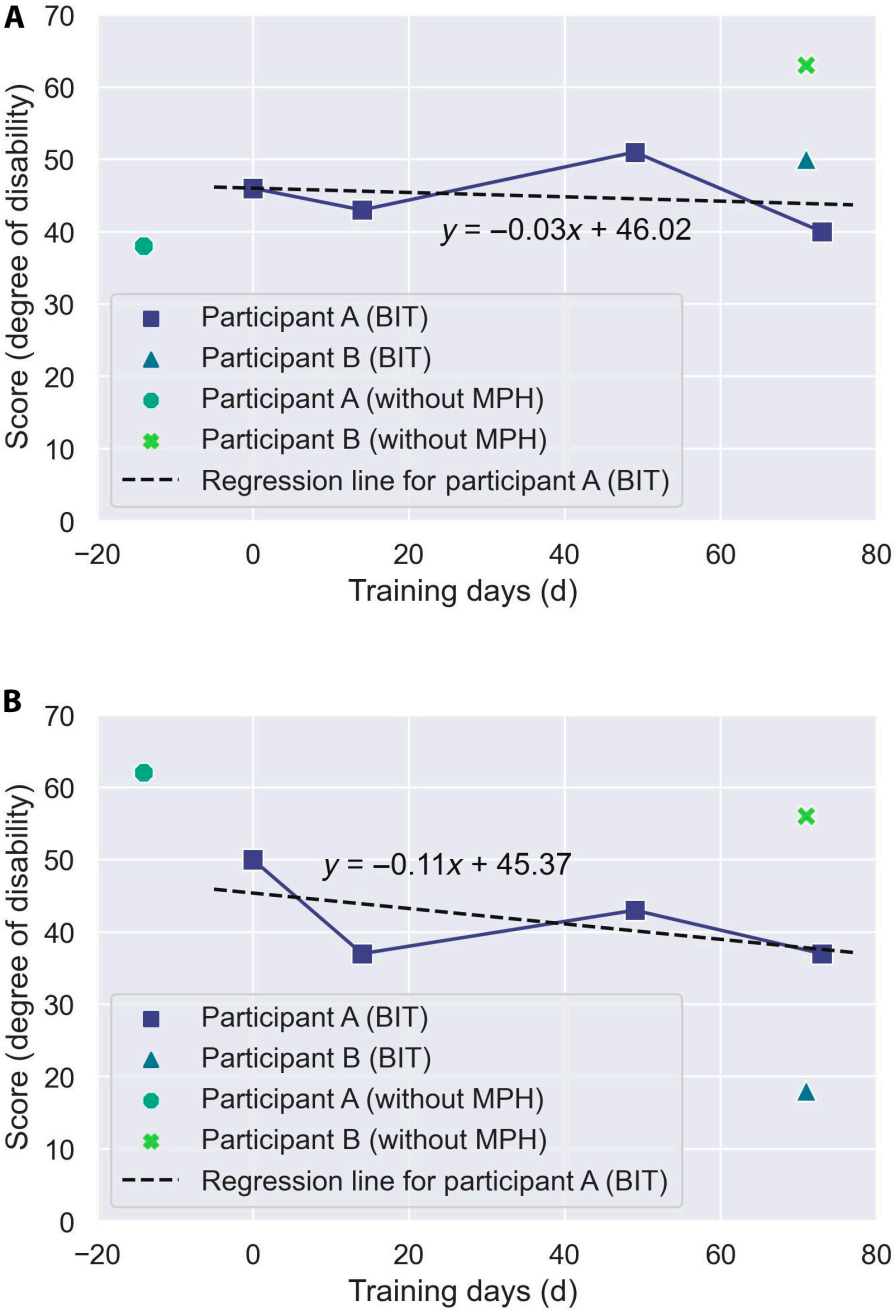
Results of the questionnaire regarding the degree of disability. “BIT” indicates the level of disability when wearing the BIT-UEC-Hand. “Without MPH” indicates the level of disability when not wearing the MPH. To facilitate comparison, the regression function of the “Participant A (BIT)” data is shown as a dotted line. Notably, higher values denote higher levels of disability. Day 0 is the day that the participant started using the MPH in each household. (A) Degree of disability in daily life. (B) Degree of disability at workplace.

The DASH results are shown in Fig. 6, and the results of the additional questionnaire are shown in Table 2(a). Notably, a higher DASH score indicates a higher degree of disability.

The summary of DASH results is presented in Fig. 6. Fig. 6A illustrates the level of disability in daily life, while Fig. 6B demonstrates the extent of disability experienced at work. “BIT” indicates the disability level when wearing the BIT-UEC-Hand, whereas “without MPH” signifies the disability level without utilizing the MPH. The DASH outcomes indicate a gradual improvement trend in daily life disability with continuous use of the BIT-UEC-Hand at home, as depicted by participant A (BIT) in Fig. 6B. Conversely, the degree of work-related disability exhibited a more pronounced improvement when using the BIT-UEC-Hand both at home and at work, as indicated by participant A (BIT) in Fig. 6B. This discrepancy can be comprehended by observing the linear regression coefficient of participant A’s (BIT) results, which stands at −0.03 for disability/symptom and −0.11 for work. Notably, the slope’s absolute value is larger for work-related disability. The restrained improvement trend in daily life disability could be attributed to both participants being single-handed amputees, capable of performing most daily activities using their nonaffected hand side [41]. Moreover, Fig. 4A underscores that utilizing the BIT-UEC-Hand at home enhances task performance, implying that enhancements in task execution with the MPH may more effectively mitigate workplace disability than everyday life disability. Typically, in MPH research, task evaluations often concentrate on replicating daily living activities, akin to this study. However, incorporating task assessments linked to participants’ work activities could facilitate a more detailed examination of disability improvement effects.

**Fig. 7.**
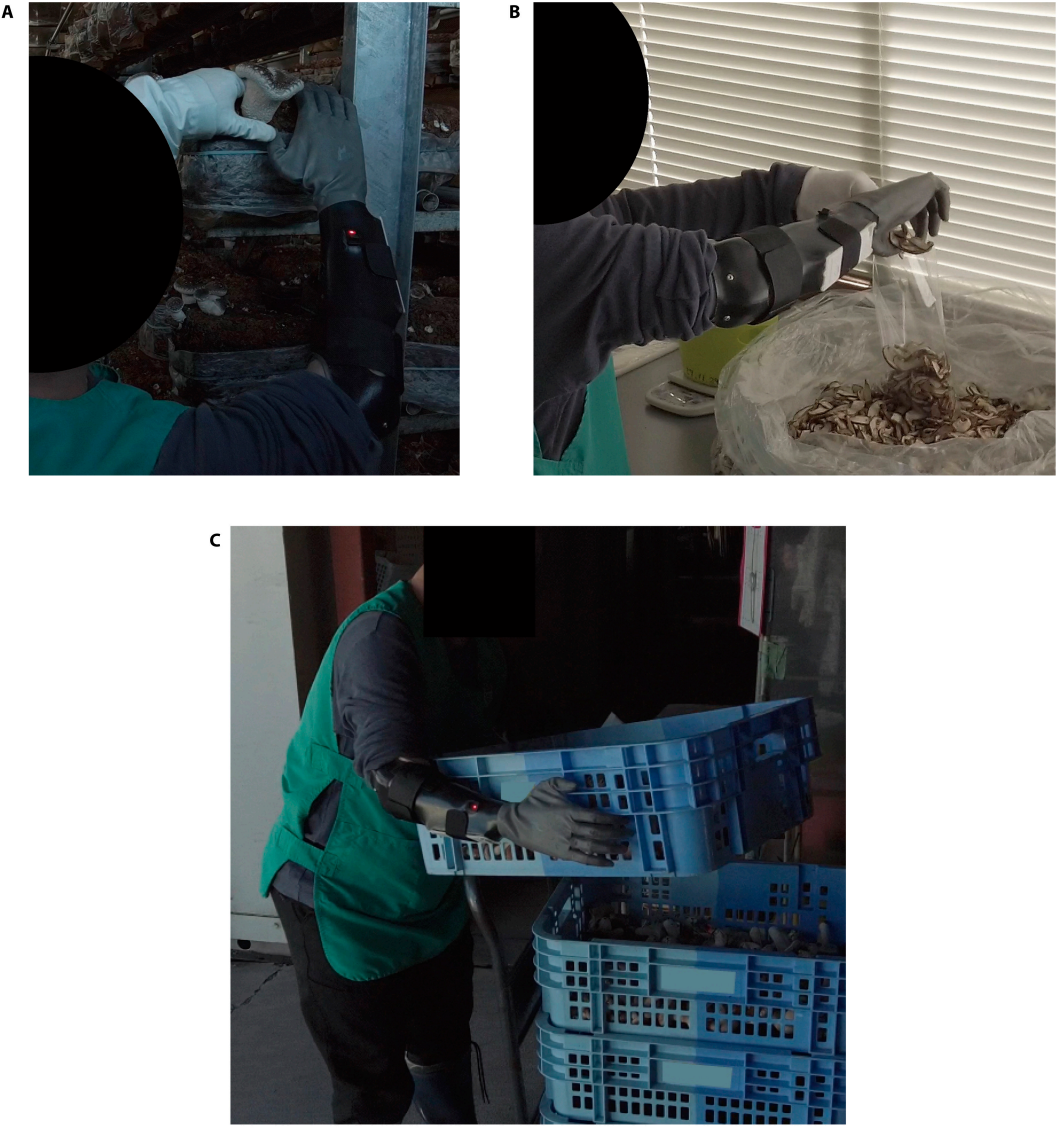
Actual examples of participants working with the BIT-UEC-Hand. (A) Participant A harvests shiitake mushrooms with the unaffected hand while holding a mushroom bed with the BIT-UEC-Hand (Video S1). (B) Participant A grabs dried mushrooms with the BIT-UEC-Hand and places them in a bag (Video S2). (C) Participant A carries a case containing shiitake mushrooms using both hands (Video S3).

Moreover, the efficacy of the MPH could substantially influence occupational choices. In Japan, the prevalent public payment system often supplies a single-DOF MPH, such as MyoBock (Ottobock) [42]. However, based on the outcomes of this study, actively providing a more functionally versatile multi-DOF MPH could ensure equitable occupational opportunities. Participant A’s utilization of the BIT-UEC-Hand in the workplace (Fig. 7) underscores its effectiveness. Additionally, during the use of the BIT-UEC-Hand at work, the unaffected hand served as support for holding a bag, while the MPH assisted in grasping dried shiitake mushrooms and placing them in the bag (Fig. 7B). The existing payment system assumes the MPH functions as an assisting hand for individuals with unilateral amputations, yet the findings from this study suggest a potential need for revising this assumption. Since limited studies have explored the application of multi-DOF MPHs in workplaces, as demonstrated in this research, future investigations in this domain will focus on validating their efficacy across various workplace settings, encompassing a wider array of multi-DOF MPH types, control methods, and participant profiles.

One notable outcome involves comparing the “without MPH” data, representing results without MPH usage, with the “BIT” data, signifying outcomes while using the BIT-UEC-Hand. Across this comparison, barring participant A’s results in daily living activities, employing the BIT-UEC-Hand yielded a lower disability level. The discrepancy observed in participant A’s disability level during daily living activities while using the BIT-UEC-Hand stemmed from differing perceptions regarding “BIT” between participant A and participant B. Participant A responded to survey queries assuming primary utilization of the BIT-UEC-Hand for each activity, whereas participant B envisaged supplementary use of the BIT-UEC-Hand alongside their unaffected hand. As previously noted, most daily activities can be executed using a healthy hand, whereas participant A, as depicted in Fig. 7, engages in a job necessitating both hands. Hence, it is plausible to infer that within such an assessment framework, workplace-related disability levels, particularly tasks requiring bilateral hand involvement, exhibited more improvement with the use of the BIT-UEC-Hand compared to “without MPH”.

The results of the additional questionnaire also revealed the limitations of the current pattern recognition control. The number of usable motions was 3 or 4, including resting motions. Even if the number of sensors used was 3 channels, many studies reported that more than 10 movements could be recognized with a high discrimination rate [7]. The performance difference between the laboratory and clinical environments was remarkable. Our findings support the results of previous studies, which indicated that the identification rate was not proportional to the actual usability of the control system [43,44].

Regarding relearning, both participants A and B answered that they needed to relearn frequently. However, participant A, who had been a user of a pattern-recognition-controlled MPH before the experiment, did not consider relearning as a burden, indicating individual differences in recognition. Participant B, however, felt that it was relatively burdensome. Therefore, it would be essential to conduct research to reduce the amount and burden of relearning to ease the entry barrier to pattern recognition control.

It is important to note that these results are specific to pattern recognition control using ANN. In this study, we employed ANN, which have been proven effective in previous research. However, there are other classical pattern recognition control methods that can be implemented on microcontrollers. Examples include studies using linear discriminant analysis and support vector machines [45,46]. Future work should investigate the long-term performance of these methods and compare them with the results of the ANN used in this study.

### Functioning of the 5 fingers

In this section, our examination focused on assessing the complete functionality of the 5 fingers. The potential advantages of the BIT-UEC-Hand, developed in this study, over conventional single-DOF MPHs are outlined as follows:

1. realization of various hand movements
2. stable grasping by increasing the contact points because the 5 fingers grip along the object [17]
3. the fingers in the way during precision grasping can be flexed and extended to enable grasping in tight spaces
4. enables the palm to be extended and to hold things down [47]

We considered that advantage 1 had been partially realized. The reasons for the partial realization are due to the following limitations:

• Limitations owing to the fingers’ range of motion and the hand’s shape. The range of motion of the fingers and the handshape limited the feasible hand movements. In particular, according to Cipriani et al. [48], 35% of daily activities are accounted for by a power grip, 30% by a precision grip, and 20% by a lateral pinch. However, the BIT-UEC-Hand cannot perform a lateral pinch owing to the limitations of its hands, which is a major functional limitation. Therefore, in future research, the problems caused by the inability to perform lateral pinch should be further examined in terms of the magnitude of compensatory movements associated with the inability. Another limitation common to many MPHs is the limitation between the reachable space of the thumb. Cotugno et al. [49] reported that the kinematics of the robot’s thumb did not equally cover the reachable space of the human thumb, favoring precision grasping movements. Furthermore, the BIT-UEC-Hand has only 1 DOF for the thumb, which considerably restricts the reachable area and, thus, the feasible hand movements.
• Limitation by the number of distinguishable patterns. Even if any movement can be realized due to the performance of the hand, its function is limited if it cannot be controlled. In this study, the questionnaire given to the participants revealed that stable control was possible in 3 to 4 movements, including rest. Furthermore, the participants could grasp a small pipe, a washer, and a metal sphere by combining a 3-finger pinch in the UEFT. Although this partially enabled a variety of hand motions, there is still a limitation in the number of patterns. To solve this problem, it is necessary to introduce adaptive identification methods that promote stable identification and evaluate the measurement stability when the EMG sensor is placed in the socket.

We considered that advantage 2 had been realized because the participants could grasp various objects in the UEFT. For example, the participants could approach different-sized cubes, rectangles, spheres, and cylinders with multiple fingers and stably grasp them at numerous contact points.

**Fig. 8.**
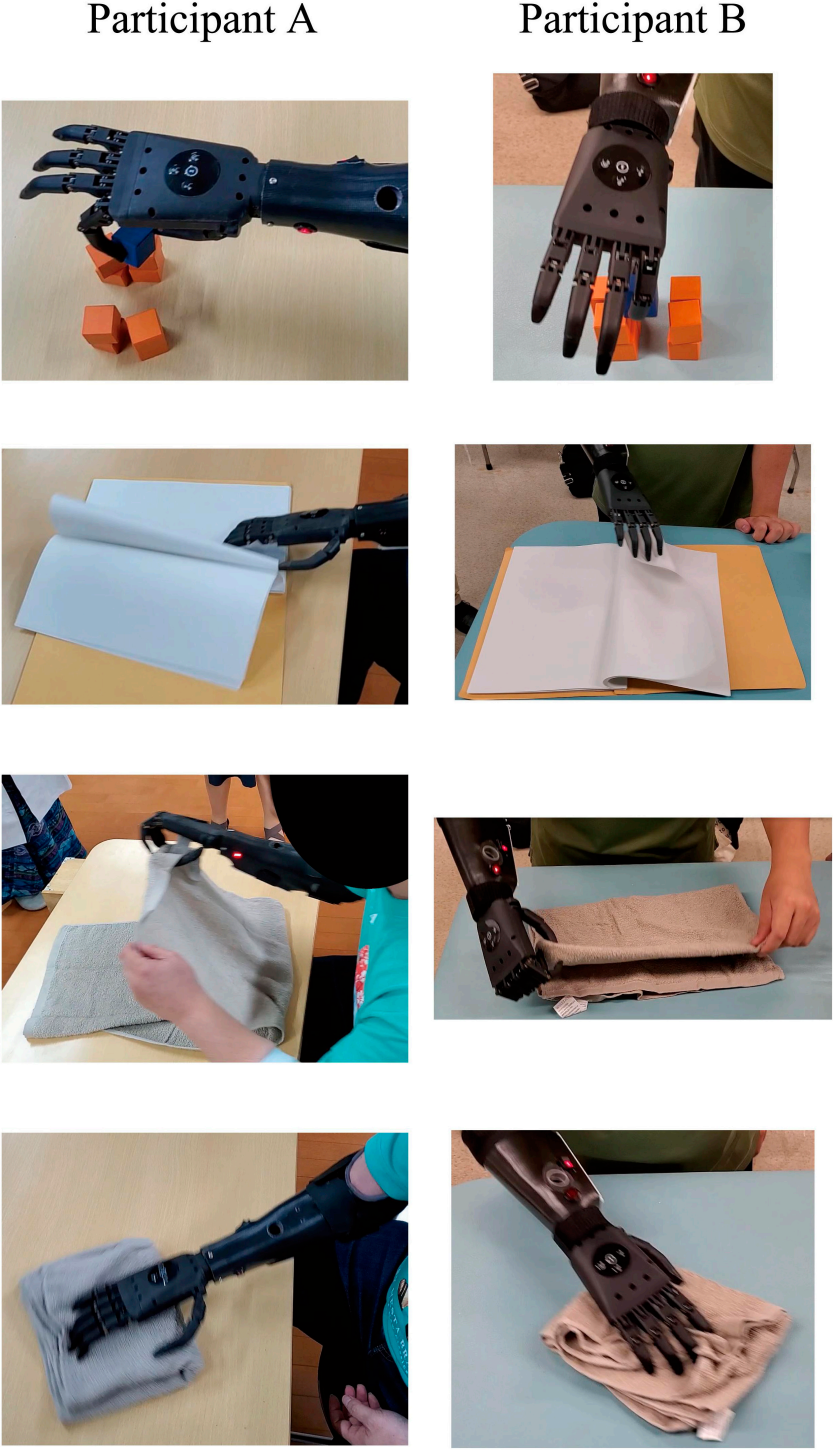
Each participant performing additional tasks. From top to bottom: Grasping a block in a narrow space, folding a page, folding a towel, and wiping a desk with a towel.

As advantages 3 and 4 could not be verified based only on this research’s task evaluation, we performed additional tasks to confirm their feasibility. As a result, as shown in Fig. 8, folding a towel to wipe a table, grasping a block in a narrow space, and turning a page were possible and achieved. Such movements are impossible with a single-DOF MPH, in which the thumb is fixed in the opposing position; therefore, this is an example of utilizing the 5-finger function.

Table 2(b) shows the range of applications of the multi-DOF MPH developed in this study.

### Toward a cyborg

In this subsection, we addressed the existing gaps identified within the outcomes of this study concerning the implementation of MPHs as cyborgs. The term “cyborg” originates from the fusion of “cybernetic”, denoting the interaction between animals and machines, and “organism”, representing a life-form, an organism, a living entity, or an organ. Clynes and Kline [50] initially defined a cyborg as an exogenously expanded tissue complex operating as an unconsciously integrated homeostatic system. However, in contemporary research, “cyborg” has evolved to signify the fusion of the human body with machines [51,52], a definition adopted within this study as well. Within MPHs, the pivotal aspect of this fusion involves synchronizing hand movements with the user’s motor intentions. This subsection probes into this alignment, evaluating the current extent of body integration achieved by MPHs.

As described in the study by Franzke et al., the pattern recognition control method used in the MPH in this research is more intuitive than conventional control methods. At the same time, however, a robustness problem was reported by Franzke et al. [15]. This problem is also pointed out in the “Questionnaire evaluation” section, where only 3 or 4 motions can be used stably. Furthermore, as pointed out in the “Questionnaire evaluation” section, an increase in the number of motions may cause problems with control speed and stability. Therefore, for implementation as a cyborg, it is necessary to develop a method that does not impair the stability by increasing the number of motion patterns, even under practical conditions such as those applied in this study.

In addition, the issue of comfort during operation has also been reported. Suzuki et al. [53] showed a gap between the operability of a nondisabled hand and that of a pattern-recognition-controlled MPH. The EMG signal is easily affected by noises when the muscle contraction force is low, and to prevent this, the operator must consciously continue the muscle contraction, which feels unnatural to the operator.

The interface’s smoothness emerged as an issue highlighted in Table 2(a), where both participants noted the necessity for frequent relearning when using the system. This requirement for retraining suggests that reattaching MPHs can alter myoelectric signals due to sensor position shifts compared to their preattachment state. This issue, previously documented, arises not only from electrode position alterations during prosthetic hand reattachment but also from changes in electrode conductivity due to perspiration during use. Additionally, changes in the user’s cognitive intent and muscle contraction strength contribute to this phenomenon [54,55]. These factors induce variations in the probability distribution of the target motion’s data, necessitating retraining with updated teacher data. Such a need for retraining poses a challenge to seamlessly integrating machine and body, representing a substantial hurdle in MPHs for maintaining recognition device continuity and realizing an integrated cyborg system. This study confirmed these alterations in myoelectric signals, underscoring the necessity of developing strategies to address this issue. However, it has been suggested that temporal variations in myoelectric signals might not invariably impede integration. According to Table 2(a), participant A reported no burden associated with retraining, while participant B described it as moderate. Notably, participant A, an adept user of pattern-recognition-controlled MPHs, could have adapted to the retraining process, potentially diminishing its hindrance to integrating the MPH with the body. Nevertheless, participant A indicated a capability to perform only 4 motions. Given that the burden of retraining escalates with an increased number of motions, the development of sustainable control methods remains a critical issue, even after mastering retraining for a limited number of motions.

In this study, we also obtained results that may be a stepping stone toward developing cyborgs. Participant A originally used an MPH controlled by pattern recognition, but the score did not reach its maximum value immediately in the UEFT. However, the score increased as the number of training days increased. This suggests that the participants improved their grasping skills by using the MPH in daily life and by repeating the UEFT to find the optimal grasping strategy. It is known that grasping strategies in early childhood are explored through trial-and-error learning [56]. It is suggested that such experiential learning of grasping strategies is brought about by the long-term application of MPHs. This should be further verified in future studies.

## Conclusion

In this study, we developed a 5-finger MPH with a pattern recognition function and applied it to 2 participants with an upper limb deficiency for 3 months. During the application, periodic evaluations were conducted to quantitatively assess the effect of the MPH on the user in terms of task performance and subjective disability level. The task evaluation revealed that the number of tasks that could be performed increased, but the control reliability decreased with the increased number of movements. These results support the advantage of the availability of various movements in pattern recognition control, which has been actively studied [9–11], and the accompanying decrease in control stability [15].

Additionally, the users engaged in this study, particularly participant B, who had no prior experience with pattern-recognition-controlled MPHs, surpassed the UEFT recordings of a conventional pattern-recognition-controlled MPH user, participant A, after approximately 80 d of home use. This outcome contradicts prior studies, suggesting that extensive training is not imperative for mastering pattern-recognition-controlled MPHs. Notably, our questionnaire survey during prolonged application indicated that enhancements in task performance with MPHs might more effectively alleviate workplace disability compared to everyday life. Our findings imply that Japan’s prevalent practice of providing single-DOF MPHs for daily activities might compromise equal occupational opportunities for individuals with disabilities relative to those without disabilities. However, it is essential to note the study’s limitations, including the inclusion of only 2 participants and differences in evaluation centers due to varying participant residences. Consequently, there were periods when it was not possible to conduct evaluations using the same assessment methods at the same time points. Therefore, future studies should ensure that evaluations are performed using consistent methods at the same time points during the assessment period to enable more detailed comparisons.

## Data Availability

The data used to support the findings of this study are available from the corresponding author upon reasonable request.

## Supplementary Materials

Supplementary 1 Videos S1 to S3
